# Development and Validation of an Interpretable Conformal Predictor to Predict Sepsis Mortality Risk: Retrospective Cohort Study

**DOI:** 10.2196/50369

**Published:** 2024-03-18

**Authors:** Meicheng Yang, Hui Chen, Wenhan Hu, Massimo Mischi, Caifeng Shan, Jianqing Li, Xi Long, Chengyu Liu

**Affiliations:** 1 State Key Laboratory of Digital Medical Engineering, School of Instrument Science and Engineering Southeast University Nanjing China; 2 Department of Critical Care Medicine Jiangsu Provincial Key Laboratory of Critical Care Medicine Zhongda Hospital, Southeast University Nanjing China; 3 Department of Electrical Engineering Eindhoven University of Technology Eindhoven Netherlands; 4 College of Electrical Engineering and Automation Shandong University of Science and Technology Qingdao China; 5 School of Intelligence Science and Technology Nanjing University Nanjing China; 6 School of Biomedical Engineering and Informatics Nanjing Medical University Nanjing China

**Keywords:** sepsis, critical care, clinical decision-making, mortality prediction, conformal prediction

## Abstract

**Background:**

Early and reliable identification of patients with sepsis who are at high risk of mortality is important to improve clinical outcomes. However, 3 major barriers to artificial intelligence (AI) models, including the lack of interpretability, the difficulty in generalizability, and the risk of automation bias, hinder the widespread adoption of AI models for use in clinical practice.

**Objective:**

This study aimed to develop and validate (internally and externally) a conformal predictor of sepsis mortality risk in patients who are critically ill, leveraging AI-assisted prediction modeling. The proposed approach enables explaining the model output and assessing its confidence level.

**Methods:**

We retrospectively extracted data on adult patients with sepsis from a database collected in a teaching hospital at Beth Israel Deaconess Medical Center for model training and internal validation. A large multicenter critical care database from the Philips eICU Research Institute was used for external validation. A total of 103 clinical features were extracted from the first day after admission. We developed an AI model using gradient-boosting machines to predict the mortality risk of sepsis and used Mondrian conformal prediction to estimate the prediction uncertainty. The Shapley additive explanation method was used to explain the model.

**Results:**

A total of 16,746 (80%) patients from Beth Israel Deaconess Medical Center were used to train the model. When tested on the internal validation population of 4187 (20%) patients, the model achieved an area under the receiver operating characteristic curve of 0.858 (95% CI 0.845-0.871), which was reduced to 0.800 (95% CI 0.789-0.811) when externally validated on 10,362 patients from the Philips eICU database. At a specified confidence level of 90% for the internal validation cohort the percentage of error predictions (n=438) out of all predictions (n=4187) was 10.5%, with 1229 (29.4%) predictions requiring clinician review. In contrast, the AI model without conformal prediction made 1449 (34.6%) errors. When externally validated, more predictions (n=4004, 38.6%) were flagged for clinician review due to interdatabase heterogeneity. Nevertheless, the model still produced significantly lower error rates compared to the point predictions by AI (n=1221, 11.8% vs n=4540, 43.8%). The most important predictors identified in this predictive model were Acute Physiology Score III, age, urine output, vasopressors, and pulmonary infection. Clinically relevant risk factors contributing to a single patient were also examined to show how the risk arose.

**Conclusions:**

By combining model explanation and conformal prediction, AI-based systems can be better translated into medical practice for clinical decision-making.

## Introduction

Sepsis is a life-threatening systemic illness resulting from a dysregulated host response to microbial invasion (infection) and is associated with high morbidity and mortality [1,2]. According to the most recent Global Burden of Diseases study, nearly 49 million people experience sepsis each year and approximately 11 million die from sepsis and its complications, accounting for 19.7% of all deaths worldwide [3]. In addition, a Chinese epidemiological study in 2020 showed that the incidence of sepsis in intensive care units (ICUs) was 20.6%, with a mortality rate of 35.5% [4]. Recent evidence suggests that early identification of patients who are critically ill with the potential for acute deterioration is effective in improving clinical outcomes [5]. Therefore, a pragmatic model that could help identify high-risk patients and further improve the prognosis of patients with sepsis is critical.

Recent significant increases in electronic health record data and advancements in artificial intelligence (AI) have led to rapid growth in the development of machine learning algorithms to identify patients with sepsis at high risk of in-hospital mortality [6-8]. However, there are 3 major barriers to the widespread adoption of AI models for their deployment in clinical practice: first, the lack of interpretability of AI models; second, the lack of external validation so that the model generalizability across institutions cannot be guaranteed; and third, the risk of automation bias, where users tend to rely too much on the system output rather than actively seeking information and assessing the model uncertainty.

Although numerous AI methods, especially deep learning, have demonstrated remarkable performance in medicine, surprisingly, few constructed models have been used in clinical practice, with poor interpretability being a major reason [9]. Clinicians need to understand how AI-based algorithms generate their predictions and gain insight into the precise changes in risk induced by certain factors of an individual patient [10,11]. Moreover, the majority of current sepsis mortality prediction models published are built on data from a single hospital or a uniform health care system, where the care processes are standardized or similar [6-8]. It is challenging for AI models to ensure accuracy in different hospital settings [7,12]. Therefore, another challenge in predictive modeling is how to quantify the reliability of the model predictions for new patients, especially when such data are outside the “domain” on which the model was trained [13]. Furthermore, most AI models only provide binary predictions, that is, yes or no, without assessing how reliable a prediction is [14]. However, when using AI models in high-risk environments (such as ICUs), uncertainty quantification is required to avoid unexpected model failures by gaining insight into the confidence of the predictions made by an AI algorithm [15].

To the best of our knowledge, no models have been explicitly developed to estimate the uncertainty of AI-assisted critical illness risk predictions in patients admitted to the ICU using electronic health record data. In this study, we aimed to develop and validate an AI model, called CPMORS (Conformal Predictor for Mortality Risk in Sepsis), to assess the risk of in-hospital sepsis mortality in ICU admissions. To mitigate the impact of insufficient model generalization performance and automation bias on its clinical application, we expected the model to provide confidence measures to monitor predictions and flag uncertain predictions at a customized confidence level for human intervention, as well as to provide interpretable risk factors, in this case, to improve the translation of AI-assisted sepsis prediction systems into medical practice and enable intensivists to use them in clinical decision-making.

## Methods

### Data Sources

The data used in this retrospective study were obtained from 2 different databases with different clinical information systems: Medical Information Mart for Intensive Care database-IV (MIMIC-IV; version 2.2; MetaVision system) [16] and eICU Collaborative Research Database (eICU-CRD; Philips eICU system) [17]. MIMIC-IV provided critical care data of 73,181 patients admitted to the ICUs at Beth Israel Deaconess Medical Center between 2008 and 2019. eICU-CRD is a large multicenter ICU database including more than 200,000 ICU admissions from 335 units in 208 hospitals across the United States between 2014 and 2015.

### Ethical Considerations

The MIMIC-IV and eICU-CRD were publicly available databases and were previously ethically approved by the institutional review boards at Beth Israel Deaconess Medical Center and the Massachusetts Institute of Technology in accordance with the tenets of the Declaration of Helsinki. The waiver of the requirement for informed consent was included in the institutional review board approval as all protected health information was deidentified [16,17]. The authors were granted access to the database after completing training in human research and signing a data use agreement in PhysioNet (certification number: 27252652).

### Participant Selection

In MIMIC-IV, participants were enrolled based on the sepsis-3 criteria [2], that is, known or suspected infection and a Sequential Organ Failure Assessment (SOFA) score ≥2 points. In eICU-CRD, patients with sepsis were identified according to the admission diagnosis recorded in the Acute Physiology and Chronic Health Evaluation IV data set [18]. For those with multiple ICU admissions, only the first ICU admission was included. Patients who were discharged from the ICU within 24 hours and were aged <18 years were excluded. Patients with >30% missing individual data were also excluded.

### Data Extraction and Preprocessing

Patient data from the first 24 hours after ICU admission were retrieved from the MIMIC-IV and eICU-CRD databases to predict in-hospital mortality risk. The study retrospectively collected the following data: (1) demographic characteristics, including sex, age, BMI, and ethnicity; (2) site of infection, including pulmonary and gastrointestinal infections; (3) comorbidities, including chronic kidney disease, congestive heart failure, chronic pulmonary disease, diabetes, and liver disease; (4) worst reported Glasgow Coma Scale, Acute Physiology Score III (APS III), and SOFA score; (5) vital signs, including heart rate, respiratory rate, systolic blood pressure, mean blood pressure, diastolic blood pressure, temperature, and oxygen saturation; (6) laboratory data, including pH, lactate, bicarbonate, base excess, PaO_2_, PaCO_2_, FiO_2_, PaO_2_/FiO_2_ ratio, hematocrit, hemoglobin, platelets, white blood cells count, albumin, anion gap, blood glucose, blood urea nitrogen, serum calcium, serum creatinine, serum sodium, serum potassium, international normalized ratio, prothrombin time, partial thromboplastin time, alanine transaminase, alkaline phosphatase, aspartate aminotransferase, and total bilirubin; (7) therapeutic management, including the use of vasopressors; and (8) total urine output.

We used one-hot encoding for the representation of categorical variables. For vital signs with multiple measurements during the first day, we included the maximum, minimum, mean, and SD values for analysis. For laboratory values with multiple measurements, we included the maximum and minimum values for analysis. This resulted in a total of 103 features used to train and validate AI models for in-hospital mortality risk prediction (Multimedia Appendix 1). For missing data, the BMI was imputed with the k-nearest neighbors algorithm using the demographic characteristics. For the remaining missing values, if the predictive models could not support the missing data, the mean value from the training data was used to fill the remaining missing values; if the predictive models could support the missing data, no imputation was performed. All numerical features were standardized by removing the mean and scaling to unit variance. To avoid information leakage, the preprocessing operations were derived from the training data and applied to other validation data sets.

### Model Development and Validation

**Figure 1:**
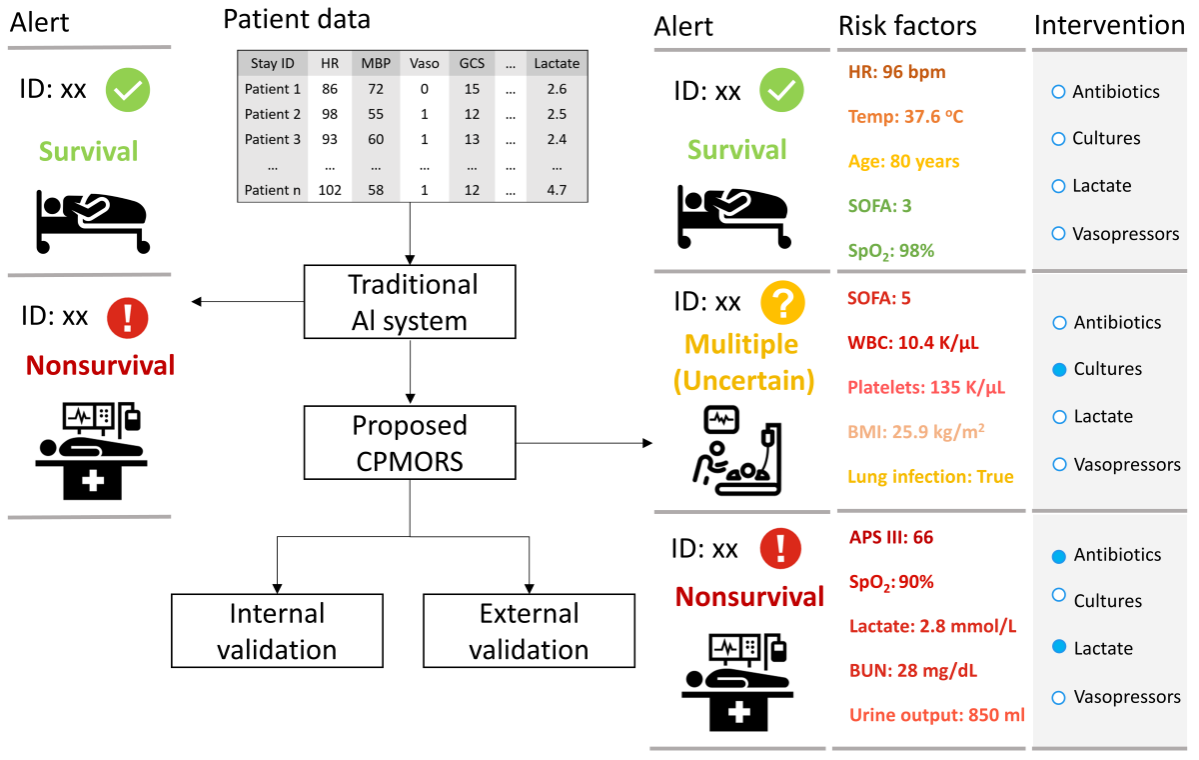
Visualization of the use of CPMORS in AI-assisted sepsis mortality risk prediction. The implication of multiple predictions is that there is insufficient information for the model to discriminate between survival and nonsurvival outcomes, which are flagged for clinician review. Risk factors are the explanations provided by the Shapley values. Risk factors in red are more serious and risk factors in green are in the normal range. AI: artificial intelligence; APS III: Acute Physiology Score III; BUN: blood urea nitrogen; CPMORS: Conformal Predictor for Mortality Risk in Sepsis; GCS: Glasgow Coma Scale; HR: heart rate; MBP: mean blood pressure; SOFA: Sequential Organ Failure Assessment; SpO_2_: oxygen saturation; WBC: white blood cell.

**Figure 2:**
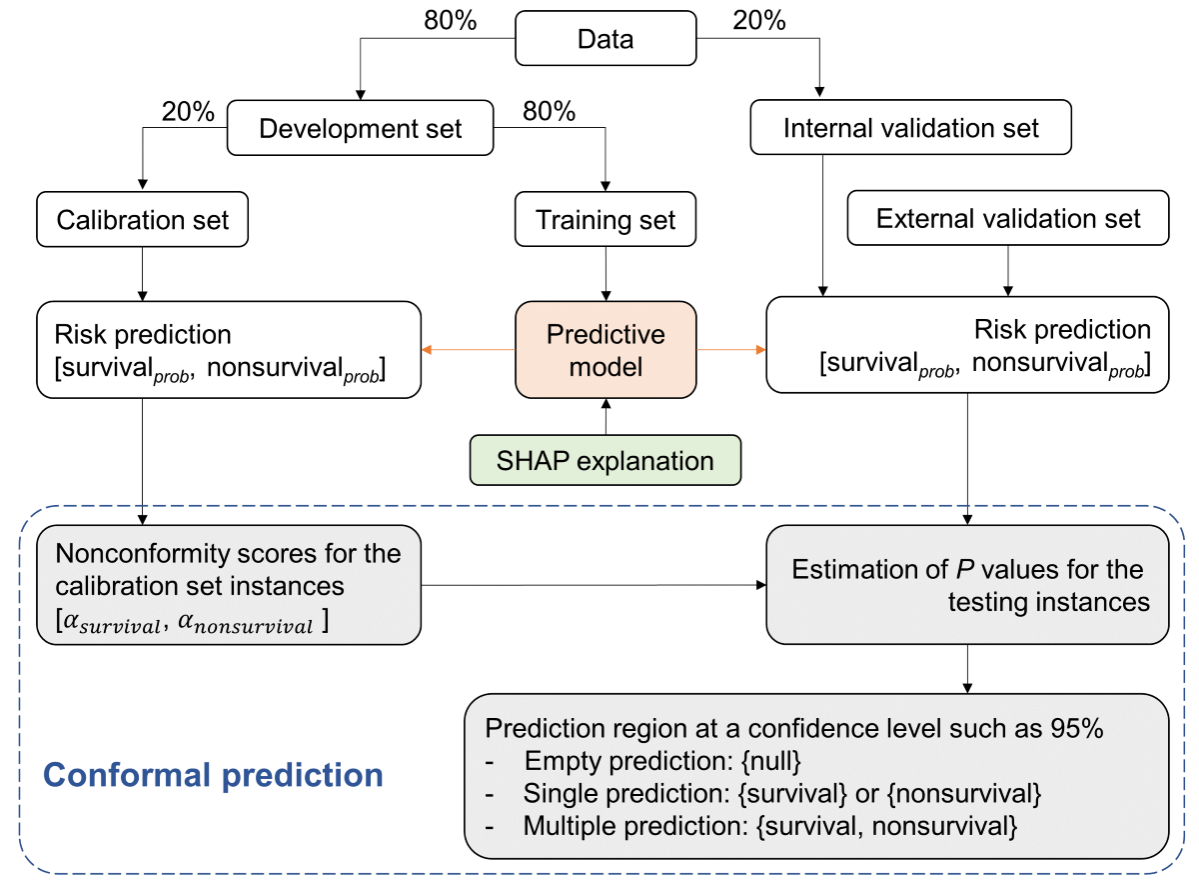
Workflow for the proposed CPMORS model. CPMORS: Conformal Predictor for Mortality Risk in Sepsis; SHAP: Shapley additive explanation.

The CPMORS model was developed to predict in-hospital mortality risk from sepsis and provide uncertain predictions and risk factors for further possible active management (Figure 1). Patients with sepsis from the MIMIC-IV database were randomly divided into a development set (n=16,746, 80%) for model training and an internal validation set (n=4187, 20%) for testing (Figure 2). Septic ICU admissions derived from the eICU-CRD database were used for external validation. The CPMORS model was constructed using the gradient boosting machines [19] prediction algorithm. Three common machine learning algorithms were also constructed for comparative purposes, including neural decision forest [20], random forest [21], and logistic regression [7]. Conventional scoring systems that have been widely used in clinical practice without machine learning, including APS III and SOFA, were also tested for comparison. Missing data were handled with a mean imputation method for neural decision forest, random forest, and logistic regression, while the gradient boosting machines did not require imputation.

To estimate the uncertainty of the model outputs at a customized or user-specified confidence level that can be set by clinicians, we used the conformal prediction (CP) framework built on top of the prediction algorithm. CP is a user-friendly paradigm for generating statistically rigorous uncertainty sets or intervals for the predictions of unknown samples that differ from the training data [15]. This approach could provide reliable predictions at a user-specified desired error rate (equal to the significance level and 1-confidence). The output of CP is a prediction region, that is, sets of labels, rather than a single value prediction (point prediction) for classification problems. In this study, the possible prediction sets were {survival} or {nonsurvival}, called a single prediction, {survival, nonsurvival}, called multiple predictions, or {null} called the empty set. Central to CP is the use of nonconformity measures to assess how dissimilar a new sample is from the data on which the model was built. In this study, we used a common nonconformity measure, that is, using the predicted probability of an example belonging to a given class to calculate the nonconformity score. The nonconformity was then used to calculate a *P* value for each possible class label when making a prediction using the conformal predictor. The *P* value represented the proportion of observations with more extreme nonconformity scores. Labels were included in the prediction set if the *P* value exceeded a user-specified desired significance level (1-confidence), such as .05 (Table 1). A multiple prediction meant that the prediction was uncertain, and the model could not distinguish between survival and nonsurvival. Empty predictions were examples where the model could not assign any label, typically meaning that the example differed from the data the model was trained on. In this study, a Mondrian CP was specifically implemented to handle classification tasks with unbalanced data [22]. It could work on a class basis to ensure the desired error rate within each class. At a higher confidence level, we got fewer error predictions but more multiple predictions (Table 2). To develop the Mondrian conformal predictor, we further split the development set into a training set (n=13,397, 80%) and a calibration set (n=3349, 20%; Figure 2). The training set was used to train the AI prediction algorithm, while the calibration set was used to construct the conformal predictor and also to tune the model hyperparameters using a Bayesian optimizer [23].

**Table 1:** Examples of the formation of the prediction set.

Confidence level	Significance level	Higher than the significance level (yes or no)		Prediction set
		*P* for survival=.37	*P* for nonsurvival=.08	
0.60	.40	No	No	{null}
0.85	.15	Yes	No	{survival}
0.95	.05	Yes	Yes	{survival, nonsurvival}

**Table 2:** Examples of predictions at 2 different confidence levels.

ID	Confidence level=85%				Confidence level=95%		
	True label^a^	Prediction set^a^	Prediction type^b^	True label^a^		Prediction set^a^	Prediction type^b^
1	0	{0, 1}	Multiple	0		{0, 1}	Multiple
2	1	{1}	Correct	1		{1}	Correct
3	1	{0}	Error	1		{0, 1}	Multiple
4	1	{0, 1}	Multiple	1		{0, 1}	Multiple
5	0	{null}	Empty	0		{1}	Error
6	0	{1}	Error	0		{1}	Error
7	0	{0}	Correct	0		{0}	Correct
8	1	{1}	Correct	1		{1}	Correct
9	1	{0}	Error	1		{0, 1}	Multiple
10	1	{1}	Correct	1		{0, 1}	Multiple

^a^Label 0=survival, label 1=nonsurvival.

^b^Correct and error predictions refer to single predictions.

To explain the model, the impact of the features on the risk output was quantified using Shapley additive explanation (SHAP) values [24] to obtain interpretability for the developed model. We provide both global feature importance from the whole population outputs and individual interpretability for a single patient output.

### Statistical Analysis

Continuous variables were expressed as median with IQR, and 2 groups were compared using the Wilcoxon rank sum test. Categorical variables were expressed as numbers and percentages and compared using chi-square tests. The discriminative performance of the model for predicting sepsis mortality was assessed using the area under the curve (AUC), and calibration was performed using calibration curves and the Brier score (the lower the better). We calculated the 95% CIs for these metrics using bootstrapping (2000 stratified bootstrap replicates).

The conformal predictor could produce multiple predictions or empty predictions in cases where it could not assign reliable single predictions. Therefore, it was not possible to directly calculate the sensitivity and specificity of the CP. To evaluate the CP framework, we assessed efficiency, defined as the proportion of all predictions that resulted in a single correct prediction, and validity (the error rate), the proportion of all predictions that did not exceed the prespecified significance level [15]. All statistical analysis and calculations were performed using R (version 4.2.2; R Foundation for Statistical Computing) and Python (version 3.8.16; Python Software Foundation).

## Results

### Patient Description

This study follows the Guidelines for Developing and Reporting Machine Learning Predictive Models in Biomedical Research [25]. A total of 20,933 adult patients from the MIMIC-IV database meeting the sepsis-3 criteria were analyzed, of whom 3457 (16.5%) were nonsurvivors. The external validation cohort from the eICU-CRD included 10,362 patients with sepsis (n=1757, 17% for nonsurvivors). Table 3 describes the baseline characteristics between survivors and nonsurvivors of patients with sepsis admitted to the ICU. Multimedia Appendix 1 shows that 93 out of 103 features were statistically different (*P*≤.05) between MIMIC-IV and eICU-CRD. In both data sets, compared with patients whose outcome was in-hospital survival, nonsurvivors were older, had a higher BMI, had more pulmonary infections, were more likely to receive vasopressors, had higher APS III and SOFA scores, and had a longer ICU stay.

**Table 3:** Baseline characteristics of the patients with sepsis following ICU^a^ admission.

Variables			MIMIC-IV^b^ (n=20,933)							eICU-CRD^c^ (n=10,362)				
			Survivors (n=17,476)		Nonsurvivors (n=3457)		*P* value		Survivors (n=8605)			Nonsurvivors (n=1757)		*P* value
Age (years), median (IQR)			67 (56-77)		71 (60-81)		<.001		66 (54-76)			70 (60-80)		<.001
Male, n (%)			10,285 (58.9)		1938 (56.1)		.002		4606 (53.5)			952 (54.2)		.62
BMI (kg/m^2^), median (IQR)			27.2 (23.8-31.7)		26.5 (23.1-31.3)		<.001		27.3 (23.1-33.2)			25.9 (22.0-31.1)		<.001
**Ethnicity, n (%)**							<.001							.97
	Asian	508 (2.9)		100 (2.9)				140 (1.6)			27 (1.5)			
	Black	1739 (10)		315 (9.1)				868 (10.1)			181 (10.3)			
	White	11.972 (68.5)		2134 (61.7)				6699 (77.9)			1361 (77.5)			
	Other	3257 (18.6)		908 (26.3)				898 (10.4)			188 (10.7)			
**Comorbidities, n (%)**														
	Chronic kidney disease	2913 (16.7)		673 (19.5)		<.001		1040 (12.1)			277 (15.8)		<.001	
	Congestive heart failure	5575 (31.9)		1332 (38.5)		<.001		1907 (22.2)			429 (24.4)		.04	
	Chronic pulmonary disease	4882 (27.9)		1055 (30.5)		.002		2015 (23.4)			415 (23.6)		.86	
	Liver disease	1209 (6.9)		487 (14.1)		<.001		96 (1.1)			79 (4.5)		<.001	
	Diabetes	5832 (33.4)		1103 (31.9)		.09		2976 (34.6)			562 (32)		.04	
Pulmonary infection, n (%)			6958 (39.8)		1983 (57.4)		<.001		4299 (50)			933 (53.1)		.02
Vasopressors, n (%)			8836 (50.6)		2518 (72.8)		<.001		2450 (28.5)			835 (47.5)		<.001
Urine output (mL), median (IQR)			1640 (1030-2465)		956 (445-1715)		<.001		1320 (689-2223)			700 (250-1351)		<.001
Glasgow Coma Scale (score), median (IQR)			15 (13-15)		15 (12-15)		.02		14 (9-15)			10 (6-14)		<.001
APS III^d^ (score), median (IQR)			46 (35-60)		66 (51-84)		<.001		51 (38-67)			71 (54-92)		<.001
SOFA^e^ (score), median (IQR)			5 (4-8)		8 (6-11)		<.001		4 (2-7)			7 (4-10)		<.001
Length of ICU stay (hours), median (IQR)			72 (44-138)		109 (58-208)		<.001		68 (43-121)			86 (46-170)		<.001

^a^ICU: intensive care unit.

^b^MIMIC-IV: Medical Information Mart for Intensive Care database-IV.

^c^eICU-CRD: eICU Collaborative Research Database.

^d^APS III: Acute Physiology Score III.

^e^SOFA: Sequential Organ Failure Assessment.

### Prediction Performance and Explanation

**Figure 3:**
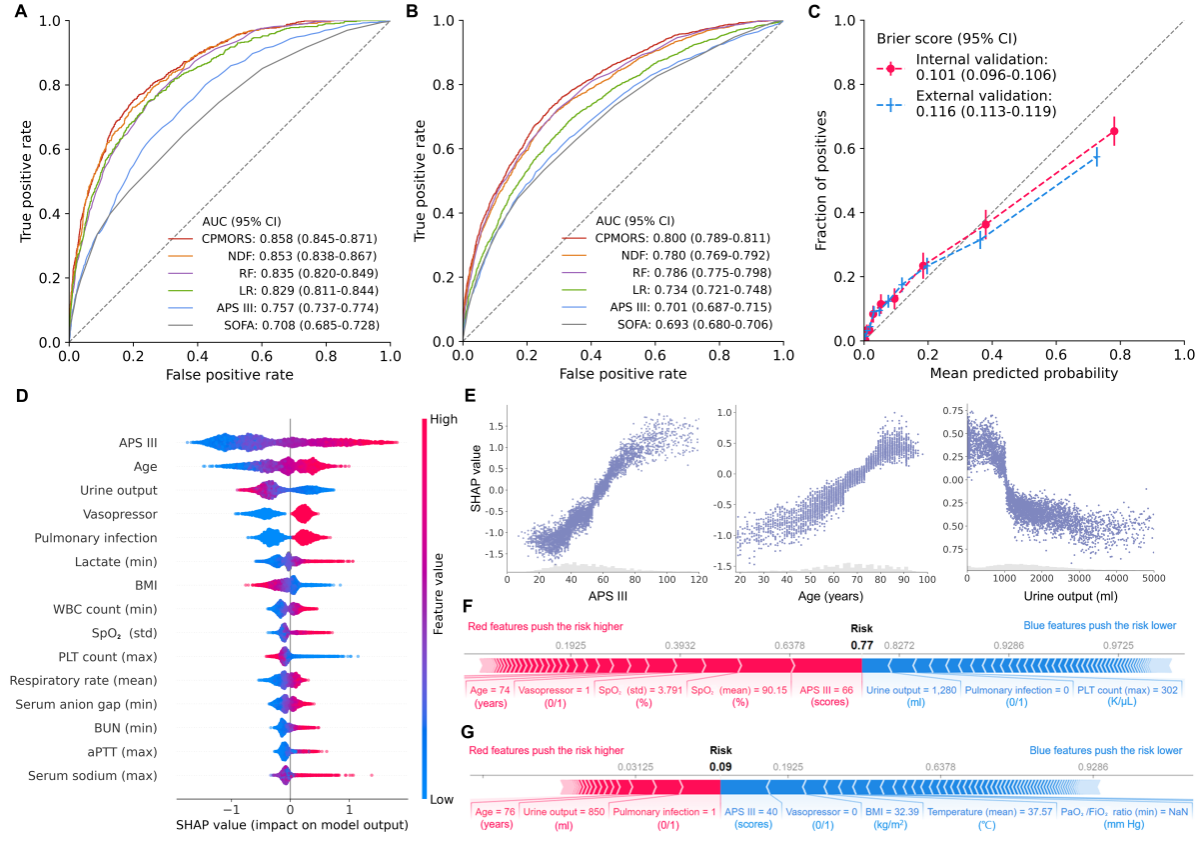
Prediction performance and explanation. (A,B) Receiver operating characteristic curves of MMIC-IV and eICU-CRD validation performance of different models in predicting sepsis mortality. (C) Calibration plots of the proposed CPMORS model. (D) Summary of the interpretability of the CPMORS. Beeswax plots show the feature importance across patients for the top 15 features, where each point indicates the feature importance value for 1 patient sample. Where multiple dots fall on the same x position, they are stacked to show density. Features with positive impact values push the risk up, while negative impact values push the risk down. Long tails indicate features that are extremely important for some patients. (E) Examples of the relationship between SHAP value and feature value. (F) Explanation of how the sepsis risk score is output for a nonsurvivor. (G) Explanation of how the sepsis risk score is output for a survivor. APS III: Acute Physiology Score III; aPTT: activated partial thromboplastin time; AUC: area under the curve; BUN: blood urea nitrogen; CPMORS: Conformal Predictor for Mortality Risk in Sepsis; eICU-CRD: eICU Collaborative Research Database; LR: logistic regression; MMIC-IV: Medical Information Mart for Intensive Care database-IV; NDF: neural decision forest; PLT: platelet; RF: random forest; SHAP: Shapley additive explanation; SOFA: Sequential Organ Failure Assessment; SpO_2_: oxygen saturation; WBC: white blood cell.

Figure 3 shows the prediction performance of the developed 6 models in terms of receiver operating characteristic curves. When tested on the internal validation population for MIMIC-IV, the AUC value obtained using the proposed CPMORS model was 0.858 (95% CI 0.845-0.871), which is significantly higher than the other 3 machine learning models ranging from 0.829 (95% CI 0.811-0.844) to 0.853 (95% CI 0.838-0.867) and the 2 scoring systems with 0.708 (95% CI 0.685-0.728) of SOFA and 0.757 (95% CI 0.737-0.774) of APS III. When externally validated on the multicenter database eICU-CRD, the performance of all models deteriorated due to interdatabase heterogeneity. Nevertheless, CPMORS still showed the best performance with AUC 0.800 (95% CI 0.789-0.811) versus other models ranging from 0.693 (95% CI 0.680-0.706) to 0.786 (95% CI 0.775-0.798). In addition, the model showed good calibration (Figure 3C), where the curves were close to the diagonal dash line. The Brier score was 0.101 (95% CI 0.096-0.106) in MIMIC-IV and 0.116 (95% CI 0.113-0.119) in eICU-CRD, indicating its high calibration ability.

The top 15 clinical features that contributed to the prediction of sepsis mortality risk are summarized in Figure 3D. The interpretable summary of the impact of the features across patients showed that a higher APS III score, older age, oliguria, the use of vasopressor, pulmonary infection, a higher lactate, a lower BMI, a higher white blood cell count, an unstable oxygen saturation, and a lower platelet count were associated with a higher mortality risk, which are consistent with the previous statistical analysis. Figure 3E shows examples of the relationship between SHAP value and feature value. Explanations representing the effects of interpretable sets of extracted features for an individual nonsurvivor and survivor are shown in Figure 3F and G. These effects explained why the model predicted a particular risk, allowing a clinician to plan appropriate interventions.

### Results for CP

**Figure 4:**
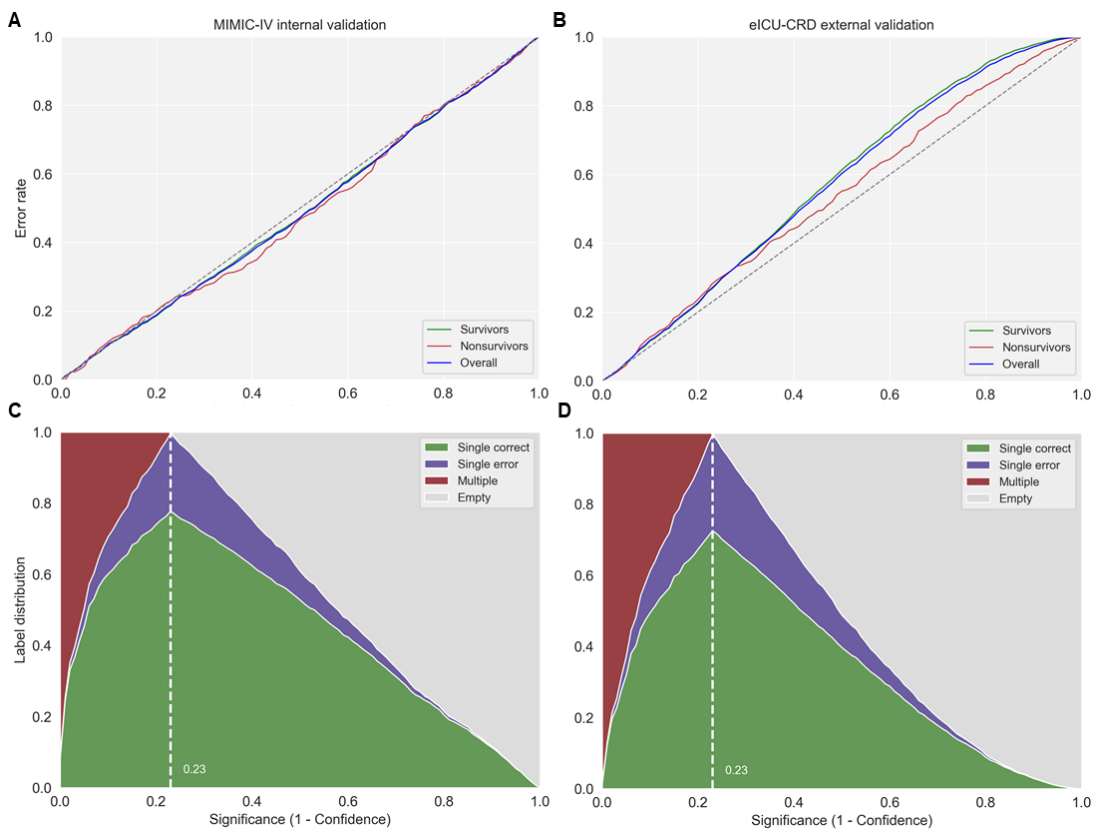
Plots of conformal prediction for the 2 validation data sets. (A,B) Calibration curves of observed prediction error (the fraction of true labels not included in the prediction region) for different prespecified significance (1-confidence) levels. (C,D) Label distribution plots at different prespecified significance levels, with the model incorporating more multiple predictions at lower significance (higher confidence) levels. The white dotted line indicates the corresponding significance level that produces the highest number of single-label predictions. Single predictions output {survival} or {nonsurvival}, multiple predictions output {survival, nonsurvival}, empty means {null}. eICU-CRD: eICU Collaborative Research Database; MIMIC-IV: Medical Information Mart for Intensive Care database-IV.

Calibration curves of the observed prediction error at a significance (1-confidence) level between 0% and 100% showed that CPMORS was well calibrated for the internal MIMIC-IV validation when the populations were from the same hospital system (Figure 4). The calibration curve for external eICU-CRD populations deviated from the ideal diagonal line but was good when the significance level was below 20%. When the conformal predictor was set at a prespecified confidence level of 90% to provide valid predictions, for the MIMIC-IV validation cohort, there were 2520 (efficiency=60.2%) single correct predictions and 1229 (29.4%) multiple predictions (Table 4). The overall validity, the percentage of error predictions (n=438) out of all predictions (n=4187), was 10.5%. In contrast, the AI model without CP made 1449 (34.6%) errors in predicting sepsis mortality risk. When the model was externally validated on a large multicenter database, that is, eICU-CRD, more multiple predictions (n=4004, 38.6%) were flagged for clinician review. In this case, the conformal predictor still produced significantly lower error rates compared to the point predictions by AI (n=1221, 11.8% vs n=4540, 43.8%). No empty predictions were made in either validation cohort.

**Table 4:** Prediction regions on the internal validation and external validation populations.

Confidence level	Prediction regions	MIMIC-IV^a^ internal validation				eICU-CRD^b^ external validation		
		Survivors (n=3496), n (%)	Nonsurvivors (n=691), n (%)	Overall (n=4187), n (%)	Survivors (n=8605), n (%)		Nonsurvivors (n=1757), n (%)	Overall (n=10,362), n (%)
**99.9%**								
	Empty	0 (0)	0 (0)	0 (0)	0 (0)		0 (0)	0 (0)
	Single error	43 (1.2)	1 (0.1)	44 (1.1)	81 (0.9)		13 (0.7)	94 (0.9)
	Single correct	868 (24.8)	145 (21)	1013 (24.2)	999 (11.6)		263 (15)	1262 (12.2)
	Multiple	2585 (73.9)	545 (78.9)	3130 (74.8)	7525 (87.4)		1481 (84.3)	9006 (86.9)
**95%**								
	Empty	0 (0)	0 (0)	0 (0)	0 (0)		0 (0)	0 (0)
	Single error	182 (5.2)	28 (4.1)	210 (5)	461 (5.4)		80 (4.6)	541 (5.2)
	Single correct	1598 (45.7)	306 (44.3)	1904 (45.5)	2704 (31.4)		616 (35.1)	3320 (32)
	Multiple	1716 (49.1)	357 (51.7)	2073 (49.5)	5440 (63.2)		1061 (60.4)	6501 (62.7)
**90%**								
	Empty	0 (0)	0 (0)	0 (0)	0 (0)		0 (0)	0 (0)
	Single error	361 (10.3)	77 (11.1)	438 (10.5)	997 (11.6)		224 (12.7)	1221 (11.8)
	Single correct	2123 (60.7)	397 (57.5)	2520 (60.2)	4285 (49.8)		852 (48.5)	5137 (49.6)
	Multiple	1012 (28.9)	217 (31.4)	1229 (29.4)	3323 (38.6)		681 (38.8)	4004 (38.6)
**85%**								
	Empty	0 (0)	0 (0)	0 (0)	0 (0)		0 (0)	0 (0)
	Single error	502 (14.4)	109 (15.8)	611 (14.6)	1454 (16.9)		322 (18.3)	1776 (17.1)
	Single correct	2390 (68.4)	470 (68)	2860 (68.3)	5171 (60.1)		1018 (57.9)	6189 (59.7)
	Multiple	604 (17.3)	112 (16.2)	716 (17.1)	1980 (23)		417 (23.7)	2397 (23.1)
**AI^c^ point prediction^d^**								
	Error	1372 (39.2)	77 (11.1)	1449 (34.6)	4316 (50.2)		224 (12.7)	4540 (43.8)
	Correct	2124 (60.8)	614 (88.9)	2738 (65.4)	4289 (49.8)		1533 (87.3)	5822 (56.2)

^a^MIMIC-IV: Medical Information Mart for Intensive Care database-IV.

^b^eICU-CRD: eICU Collaborative Research Database.

^c^AI: artificial intelligence.

^d^For comparison with the AI model with conformal prediction at a user-specified 90% confidence level, the AI point prediction without conformal prediction was made when setting the error rate in nonsurvivors as 11.1% for MIMIC-IV validation and 12.7% for eICU-CRD validation.

The top 15 features summarized by the SHAP values could not be well differentiated between the multiple predictions in survivors and nonsurvivors (Table 5 and Multimedia Appendix 2). In addition, for those patients who survived, the analysis showed that patients identified for clinician review (those with uncertain predictions) were more likely to develop new acute kidney injury after the first day of ICU admission (n=227, 22.4% vs n=428, 20.2% for MIMIC-IV; n=799, 24% vs n=875, 20.4% for eICU-CRD) and had longer ICU stays with a median of 89 (IQR 54-173) hours versus 55 (IQR 37-99) hours for MIMIC-IV, and 78 (IQR 48-141) hours versus 55 (IQR 40-91) hours for eICU-CRD, compared to the patients who were not flagged for review.

**Table 5:** Statistical analysis of the top 15 features between the single correct prediction patients and the multiple prediction patients in the MIMIC-IV^a^ validation populations.

Variables	Survivors			Nonsurvivors				
	Single correct (n=2123)	Multiple (n=1012)	*P* value	Single correct (n=397)	Multiple (n=217)	*P* value	*P* value^b^	
APS III^c^ (score), median (IQR)	39 (31-49)	55 (44-66)	<.001	78 (64-94)	58 (47-68)	<.001	.05	
Age (years), median (IQR)	64 (54-74)	72 (61-82)	<.001	71 (59-82)	75 (60-81)	.40	.57	
Urine output (mL), median (IQR)	1900 (1310-2675)	1350 (808-2065)	<.001	635 (206-1171)	1278 (845-1815)	<.001	.31	
Vasopressor, n (%)	959 (45.2)	520 (51.4)	.01	347 (87.4)	134 (61.8)	<.001	.005	
Pulmonary infection, n (%)	585 (27.6)	603 (59.6)	<.001	230 (57.9)	127 (58.5)	.89	.77	
Lactate (min) (mmol/L), median (IQR)	1.2 (1.0-1.6)	1.4 (1.1-1.9)	<.001	2.2 (1.5-3.4)	1.4 (1.1-1.9)	<.001	.72	
BMI (kg/m^2^), median (IQR)	27.8 (24.5-32.3)	26.5 (23.0-30.8)	<.001	26.8 (23.5-32.3)	25.9 (23.2-31.4)	.57	.61	
WBC^d^ count (min) (K/μL), median (IQR)	9.2 (6.6-12.1)	9.9 (6.8-14.0)	<.001	11.7 (7.0-16.4)	10.4 (6.6-14.4)	.06	.50	
SpO_2_^e^ (std) (%), median (IQR)	1.86 (1.36-2.39)	1.93 (1.34-2.55)	.08	2.45 (1.71-3.46)	2.12 (1.43-2.89)	<.001	.01	
PLT^f^ count (max) (K/μL), median (IQR)	204 (153-269)	213 (147-286)	.11	198 (128-298)	205 (137-279)	.91	.20	
Respiratory rate (mean) (bpm), median (IQR)	18 (16-21)	20 (17-22)	<.001	22 (19-25)	20 (17-23)	<.001	.22	
Serum anion gap (min) (mmol/L), median (IQR)	12 (10-14)	13 (11-16)	<.001	15 (13-19)	13 (11-16)	<.001	.34	
BUN^g^ (min) (mg/dL), median (IQR)	16 (12-24)	25 (16-41)	<.001	33 (19-50)	27 (17-42)	.006	.43	
aPTT^h^ (max) (s), median (IQR)	32.3 (28.3-39.2)	33.8 (29.0-44.5)	<.001	44.9 (33.1-64.3)	34.4 (28.4-47.1)	<.001	.83	
Serum sodium (max) (mmol/L), median (IQR)	140 (138-142)	140 (137-144)	.28	140 (136-144)	140 (136-143)	.98	.54	

^a^MIMIC-IV: Medical Information Mart for Intensive Care database-IV.

^b^Statistical analysis between survival and nonsurvival multiple predictions.

^c^APS III: Acute Physiology Score III.

^d^WBC: white blood cell.

^e^SpO_2_: oxygen saturation.

^f^PLT: platelet.

^g^BUN: blood urea nitrogen.

^h^aPTT: activated partial thromboplastin time.

## Discussion

### Principal Findings

This study proposed CPMORS, an AI model to provide reliable predictions of mortality risk for patients with sepsis admitted to the ICU. CPMORS was developed using gradient boosting machines with 103 clinical features. Internal and external validation assessed its best predictive performance in terms of discrimination and calibration compared with several other commonly used models. Global feature importance showed that a higher APS III score, older age, oliguria, the use of vasopressor, and pulmonary infection were most associated with a higher mortality risk. A Mondrian CP was built on top of the prediction algorithm to detect uncertain predictions. Compared to traditional AI models that only provide point predictions, our method could provide reliable and uncertain predictions with user-specified confidence, especially when the performance of the external validation could not be guaranteed as good as the internal validation. Interpretable information and estimates of prediction uncertainty enabled CPMORS to provide informative support for clinical decision-making.

Recent guidelines on AI-based predictive models in health care emphasize the importance of AI systems conveying their prediction confidence to users while furnishing accurate predictions and explanations [26]. In previous studies, Zhang et al [27] developed a traditional sepsis mortality risk score system that is interpretable and transparent, but the accuracy of the model is limited, with AUC in the development and validation sets of 0.789 and 0.765. Park et al [6] and Kong et al [7] demonstrated that machine learning can improve the accuracy of sepsis mortality risk prediction, but there is a lack of strong interpretable analysis. Hu et al [8] performed a detailed and interpretable analysis. However, to date, no studies have estimated the prediction uncertainty of sepsis mortality risk in patients admitted to the ICU. In the case of physicians supported by AI systems, such feedback of prediction uncertainty was invaluable in enabling them to exercise caution and not rely solely on the output of the AI system. This could serve to safeguard patients from the potential hazards of automation bias in AI systems [28]. Various methods for quantifying uncertainty, such as the Gaussian process [29], Bayesian inference [30], deep ensembles [31], and dropout [32], have been implemented in computer vision and natural language processing applications. However, their use in clinical decision-making has been limited due to the accompanying computational cost and modeling complexity. In addition, they did not provide adjustable confidence levels to suit different clinical requirements for predicting critical illness.

In this study, we used the CP framework, which is characterized by its light mathematical nature and offers a potential solution by generating prediction regions. These prediction regions are similar to the confidence intervals used in statistics, except that they are based on individual predictions rather than overall statistics [14]. The use of CP-derived prediction regions could therefore provide reliable and uncertain predictions in clinical settings. In addition, clinicians would not rely entirely on the model’s output and make immediate decisions [28]. They still need to review patients’ critical information before making decisions. Throughout this process, CPMORS could not only provide explainable risk factors so that clinicians can double-check the reasoning of AI systems and identify problematic elements [9] but could also help to achieve stratified patient management (Figure 1). The single predictions of nonsurvival made by CPMORS could be treated as red flags, meaning that clinicians should pay the most attention. The single survival predictions could be treated as green flags, meaning that the patients are relatively safe and would be monitored regularly. For the multiple predictions, we flag the yellow warnings as such unreliable cases require clinician review; it is clinically very important that AI-based decision support systems also flag uncertainty when they are not certain about the produced output [13]. In this case, CPMORS could produce much fewer false alarms to avoid exposing patients to unnecessary tests and treatments [33]. The process also enables a symbiotic interaction between AI and clinicians, which is the key to AI adoption [34]. The AI system expertly identifies cases where its predictions are highly reliable, allowing clinicians to focus their attention on challenging and less reliable predictions. Therefore, this process would not delay critical decisions. Instead, the proposed AI model could provide explainable information and also help take the pressure off clinicians by stratifying patients. Furthermore, survivors whose predictions were uncertain (multiple predictions) were more likely to develop acute kidney injury and had a longer ICU stay, which proves the validity of their clinician review. In this case, a real-time compatible software pipeline can be developed into clinical decision systems (Figure 1) by fulfilling the requirements stated in de Hond et al [26]. This includes providing an explanation of the model output, enabling end users to visually comprehend the connection between the input data and the predicted output, fostering feedback, and improvement of the predictions.

A systematic review of AI in critical care concluded that it is important to ensure the validity and reliability of predictive models across clinical settings [35]. However, it has been reported that only 6% of studies of AI applications in health care have performed external validation [36], and the sepsis mortality risk prediction model is no exception. Furthermore, previous research on external validation shows a significant decrease when the AI system is applied in different hospital settings or data sets due to several potential threats, such as population heterogeneity, differences in clinical practice, and software diversity [11,37]. Consistent with these studies, the prediction results in this study reported that all AI models deteriorated when externally validated on eICU-CRD, with the AUC of CPMORS decreasing from 0.858 to 0.800. Although transfer learning (TL) methods have been proposed to improve the model generalization ability, additional requirements are needed. For example, additional labeled target data are required for supervised TL (fine-tuning) [38] or many unlabeled data for unsupervised TL (domain adaptation) [39]. Although model performance can be improved, uncertainty estimation is not guaranteed. In this study, the proposed CPMORS did not require target data, could provide confidence measures, and was mathematically proven to be valid, which were important for application across hospital settings. For a given confidence level, the conformal predictor provided a prediction interval within which the true value should lie with a probability of the given confidence, that is, the true class is in the prediction set. Therefore, CPMORS did not aim to directly address the problem of insufficient model generalization performance by simply improving the model’s accuracy, as it is impossible for the model to achieve 100% accuracy. CPMORS could help to mitigate the risk of the model generalizability issue from another perspective, by flagging those patients whose predictions are uncertain due to dissimilarity to the training samples and reminding clinicians to check. In addition, CP can also help to detect systematic differences, as shown in Olsson et al [15] for the diagnosis of prostate cancer.

However, we should carefully consider the trade-off between producing single predictions and multiple predictions, as the number of uncertain predictions should be kept limited to avoid overloading clinicians and creating an unmanageable situation. We can see that the proportions of single predictions, empty predictions, and multiple predictions can vary for different levels of significance (1-confidence; Figure 4). To increase confidence in the estimates, a low level of significance must be set, but this would generate an increase in the number of multiple predictions (uncertain predictions). The findings of this study indicate that, when the prespecified confidence level is set to 90%, the CPMORS provides a prediction region around the point prediction that contains the true label with a 90% probability. Our results show that, for the MIMIC-IV and eICU-CRD data sets, CPMORS generated a total of 217 (31.4%) and 681 (38.8%) multiple predictions for nonsurvivors, respectively, and 1012 (28.9%) and 3323 (38.6%) multiple predictions for survivors, respectively (Table 4). The implication of the multiple predictions in this study is that there is insufficient information for the model to discriminate between survival and nonsurvival outcomes. This is demonstrated by the inability to effectively discriminate the top 15 features between the multiple predictions of survivors and nonsurvivors (Table 5 and Multimedia Appendix 2), which reminds us that new information from patients should be provided to arrive at a single correct prediction.

### Limitations

This study has several limitations. First, although we have demonstrated this model can help to mitigate the impact of insufficient model generalization performance and automation bias by providing the uncertainty estimation, we still need further studies to collect weekly or monthly statistics of uncertainty rates so that we can set a balanced confidence level for more efficient patient management. Second, more advanced nonconformal functions should be tested to achieve smaller prediction regions and higher proportions of reliable single predictions. Third, this work was not designed to directly address generalizability and bias issues, although it touched upon them. Future work is needed to solve these 2 issues more thoroughly. Nevertheless, we provided an experimental example to describe how a combination of model interpretability and CP could work to assist clinicians in predicting sepsis mortality in ICU admissions. Conformal predictors are built on top of the underlying prediction algorithm; therefore, the framework can be applied to all prediction algorithms and other predictive tasks in critical illness prediction.

### Conclusions

In summary, this study presents the development and validation of an AI model, called CPMORS, for predicting sepsis mortality risk in patients who are critically ill. CPMORS emerges as the most effective model among all the predictive models tested in this study. Importantly, CPMORS offers interpretability, allowing for transparency in the prediction-making process. The internal and external validation procedures demonstrate the ability of CPMORS to make reliable predictions and flag uncertain predictions. These findings suggest that the integration of model explanation and CP can enhance the clinical applicability of AI-assisted systems, thereby facilitating clinical decision-making in the context of sepsis management.

## Appendix

Multimedia Appendix 1 Statistical analysis of variables between Medical Information Mart for Intensive Care database-IV and eICU Collaborative Research Database.

Multimedia Appendix 2 Statistical analysis of the top 15 features between the single correct prediction patients and the multiple prediction patients in the eICU Collaborative Research Database validation populations.

## Abbreviations

AI: artificial intelligence
APS III: Acute Physiology Score III
AUC: area under the receiver operating characteristic curve
CPMORS: Conformal Predictor for Mortality Risk in Sepsis
CP: conformal prediction
eICU-CRD: eICU Collaborative Research Database
ICU: intensive care unit
MIMIC-IV: Medical Information Mart for Intensive Care database-IV
SOFA: Sequential Organ Failure Assessment
SHAP: Shapley additive explanation
TL: transfer learning

## Data Availability

The data sets and code generated and analyzed during this study are available on the PhysioNet website [40,41] and GitHub repository [42], respectively.
