# Supplementary material for: Development and Validation of an Interpretable Conformal Predictor to Predict Sepsis Mortality Risk: Retrospective Cohort Study
Source: J Med Internet Res. 2024 Mar 18;26:e50369. doi: 10.2196/50369 (PMC10985608; doi:10.2196/50369)
Supplement: Multimedia Appendix 1 [file jmir_v26i1e50369_app1.docx]

**Multimedia Appendix 1**

Table 1. Statistical analysis of variables between MIMIC-IV and eICU-CRD

| **Variables^a^** | **MIMIC-IV (n=20933)** | **eICU-CRD (n=10362)** | ***P*** |
| --- | --- | --- | --- |
| Male (0/1) | 12223 (58.4) | 5558 (53.6) | <.001 |
| Ethnicity (0/1) |  |  | <.001 |
| White | 14106 (67.4) | 8060 (77.8) |  |
| Black | 2054 (9.8) | 1049 (10.1) |  |
| Asian | 608 (2.9) | 167 (1.6) |  |
| Other | 4165 (19.9) | 1086 (10.5) |  |
| Comorbidities (0/1) |  |  |  |
| CKD | 3586 (17.1) | 1317 (12.7) | <.001 |
| CHF | 6907 (33.0) | 2336 (22.5) | <.001 |
| CPD | 5937 (28.4) | 2430 (23.5) | <.001 |
| Liver | 1696 (8.1) | 175 (1.7) | <.001 |
| Diabetes | 6935 (33.1) | 3538 (34.1) | .07 |
| Pulmonary infection (0/1) | 8941 (42.7) | 5232 (50.5) | <.001 |
| Gastrointestinal infection (0/1) | 5061 (24.2) | 1645 (15.9) | <.001 |
| Vasopressor (0/1) | 11354 (54.2) | 3285 (31.7) | <.001 |
| Age (years) | 67 (56-78) | 66 (55-77) | <.001 |
| BMI (kg/m^2^) | 27.1 (23.7-31.6) | 27.1 (22.9-32.9) | .26 |
| GCS (score) | 15 (13-15) | 14 (9-15) | <.001 |
| APS III (score) | 49 (37-64) | 54 (39-71) | <.001 |
| SOFA (score) | 6 (4-8) | 5 (3-7) | <.001 |
| Urine output (ml) | 1535 (915-2370) | 1210 (600-2100) | <.001 |
| Heart rate (min) (bpm) | 71 (61-82) | 77 (66-89) | <.001 |
| Heart rate (max) (bpm) | 105 (91-120) | 114 (99-130) | <.001 |
| Heart rate (mean) (bpm) | 86 (76-98) | 94 (82-105) | <.001 |
| Heart rate (std) (bpm) | 8.2 (5.9-11.2) | 8.9 (6.2-12.7) | <.001 |
| SBP (min) (mm Hg) | 86 (78-96) | 82 (72-95) | <.001 |
| SBP (max) (mm Hg) | 144 (131-160) | 142 (127-160) | <.001 |
| SBP (mean) (mm Hg) | 112 (104-123) | 109 (101-121) | <.001 |
| SBP (std) (mm Hg) | 13.7 (10.8-17.3) | 14.2 (10.8-18.5) | <.001 |
| DBP (min) (mm Hg) | 44 (38-50) | 43 (36-51) | <.001 |
| DBP (max) (mm Hg) | 84 (73-97) | 85 (73-99) | .01 |
| DBP (mean) (mm Hg) | 60 (54-66) | 60 (54-67) | .26 |
| DBP (std) (mm Hg) | 9.1 (7.0-12.0) | 9.5 (7.1-12.6) | <.001 |
| MAP (min) (mm Hg) | 57 (50-63) | 56 (48-65) | .83 |
| MAP (max) (mm Hg) | 100 (90-113) | 99 (87-113) | <.001 |
| MAP (mean) (mm Hg) | 75 (69-81) | 74 (68-82) | .002 |
| MAP (std) (mm Hg) | 10.1 (7.9-13.2) | 10.1 (7.8-13.3) | .25 |
| Respiratory rate (min) (bpm) | 12 (10-15) | 14 (11-17) | <.001 |
| Respiratory rate (max) (bpm) | 28 (24-32) | 30 (25-37) | <.001 |
| Respiratory rate (mean) (bpm) | 19.2 (16.8-22.2) | 21.2 (18.2-24.8) | <.001 |
| Respiratory rate (std) (bpm) | 3.9 (2.9-4.7) | 3.9 (2.9-5.4) | <.001 |
| Temperature (min) (℃) | 36.4 (36.0-36.7) | 36.4 (36.0-36.7) | .41 |
| Temperature (max) (℃) | 37.4 (37.0-38.0) | 37.5 (37.1-38.4) | <.001 |
| Temperature (mean) (℃) | 36.9 (36.6-37.2) | 36.9 (36.6-37.3) | <.001 |
| Temperature (std) (℃) | 0.38 (0.24-0.62) | 0.45 (0.27-0.70) | <.001 |
| SpO2 (min) (%) | 92 (90-95) | 91 (86-94) | <.001 |
| SpO2 (max) (%) | 100 (100-100) | 100 (99-100) | <.001 |
| SpO2 (mean) (%) | 97.3 (95.8-98.6) | 96.7 (95.2-98.2) | <.001 |
| SpO2 (std) (%) | 1.92 (1.37-2.55) | 2.13 (1.43-3.04) | <.001 |
| pH (min) | 7.31 (7.24-7.37) | 7.32 (7.23-7.40) | <.001 |
| pH (max) | 7.42 (7.37-7.46) | 7.40 (7.35-7.45) | <.001 |
| Lactate (min) (mmol/L) | 1.4 (1.0-2.0) | 1.5 (1.0-2.2) | <.001 |
| Lactate (max) (mmol/L) | 2.3 (1.5-3.7) | 2.5 (1.5-4.2) | <.001 |
| Bicarbonate (min) (mmol/L) | 21 (18-24) | 21 (17-24) | .006 |
| Bicarbonate (max) (mmol/L) | 24 (21-27) | 25 (22-28) | <.001 |
| Base excess (min) (mmol/L) | -3.0 (-7.0-0.0) | -2.7 (-8.0-1.4) | <.001 |
| Base excess (max) (mmol/L) | 0.0 (-2.0-3.0) | 0.0 (-4.2-3.3) | <.001 |
| PaO2 (min) (mm Hg) | 66 (41-98) | 71 (59-91) | <.001 |
| PaO2 (max) (mm Hg) | 171 (92-345) | 113 (80.60-179) | <.001 |
| PaCO2 (min) (mm Hg) | 35 (31-41) | 34.50 (29-41) | <.001 |
| PaCO2 (max) (mm Hg) | 47 (40-55) | 41.30 (34-52.50) | <.001 |
| PaO2/FiO2 ratio (min) | 153.9 (90.0-245.0) | 161.5 (95.0-248.0) | <.001 |
| PaO2/FiO2 ratio (max) | 298.6 (204.0-398.0) | 247.6 (170.0-338.1) | <.001 |
| FiO2 (min) (%) | 50 (40-50) | 40 (30-50) | <.001 |
| FiO2 (max) (%) | 60 (50-100) | 60 (36-100) | <.001 |
| HCT (min) (%) | 28.9 (24.7-33.5) | 29.7 (25.4-34.3) | <.001 |
| HCT (max) (%) | 33.7 (29.8-38.3) | 35.0 (30.2-40.1) | <.001 |
| Hb (min) (g/dL) | 9.5 (8.1-11.1) | 9.7 (8.2-11.2) | <.001 |
| Hb (max) (g/dL) | 11.1 (9.7-12.6) | 11.4 (9.8-13.2) | <.001 |
| PLT count (min) (K/μL) | 161 (109-228) | 167 (110-235) | .004 |
| PLT count (max) (K/μL) | 204 (145-280) | 214 (147-299) | <.001 |
| WBC count (min) (K/μL) | 9.7 (6.7-13.4) | 10.8 (7.1-15.3) | <.001 |
| WBC count (max) (K/μL) | 13.8 (9.8-18.9) | 16.0 (11.0-22.5) | <.001 |
| Albumin (min) (g/dL) | 3.0 (2.6-3.5) | 2.4 (2.0-2.9) | <.001 |
| Albumin (max) (g/dL) | 3.2 (2.7-3.6) | 2.8 (2.3-3.3) | <.001 |
| Serum anion gap (min) (mmol/L) | 13.0 (11.0-15.0) | 10.0 (7.0-12.8) | <.001 |
| Serum anion gap (max) (mmol/L) | 16.0 (14.0-20.0) | 13.6 (10.7-17.0) | <.001 |
| BUN (min) (mg/dL) | 21 (13-35) | 23 (14-37) | <.001 |
| BUN (max) (mg/dL) | 25 (17-43) | 31 (20-50) | <.001 |
| Serum calcium (min) (mmol/L) | 8.0 (7.4-8.5) | 7.8 (7.2-8.3) | <.001 |
| Serum calcium (max) (mmol/L) | 8.5 (8.0-9.0) | 8.6 (8.0-9.1) | <.001 |
| Serum chloride (min) (mmol/L) | 102 (98-106) | 101 (96-105) | <.001 |
| Serum chloride (max) (mmol/L) | 107 (102-111) | 107 (102-111) | <.001 |
| Serum creatinine (min) (mg/dL) | 1.00 (0.70-1.60) | 1.09 (0.73-1.83) | <.001 |
| Serum creatinine (max) (mg/dL) | 1.20 (0.90-2.10) | 1.50 (0.97-2.61) | <0.001 |
| Glucose (min) (mg/dL) | 111 (93-134) | 97 (80-118) | <.001 |
| Glucose (max) (mg/dL) | 148 (119-201) | 174 (136-240) | <.001 |
| Serum sodium (min) (mmol/L) | 137 (134-140) | 136 (132-139) | <.001 |
| Serum sodium (max) (mmol/L) | 140 (137-143) | 140 (137-143) | .11 |
| Serum potassium (min) (mmol/L) | 3.9 (3.5-4.3) | 3.6 (3.3-4.1) | <.001 |
| Serum potassium (max) (mmol/L) | 4.5 (4.1-5.0) | 4.4 (4.0-4.9) | <.001 |
| INR (min) | 1.20 (1.10-1.50) | 1.30 (1.10-1.60) | <.001 |
| INR (max) | 1.40 (1.20-1.70) | 1.33 (1.13-1.90) | .03 |
| PT (min) (seconds) | 13.7 (12.3-16.0) | 15.3 (13.4-18.5) | <.001 |
| PT (max) (seconds) | 15.1 (13.2-18.8) | 16.0 (13.7-21.0) | <.001 |
| aPTT (min) (seconds) | 29.5 (26.2-34.2) | 32.9 (28.2-38.5) | <.001 |
| aPTT (max) (seconds) | 33.7 (28.9-44.7) | 34.6 (29.5-43.7) | .05 |
| ALT (min) (IU/L) | 27 (16-58) | 24 (15-44) | <.001 |
| ALT (max) (IU/L) | 30 (17-72) | 29 (18-57) | <.001 |
| ALP (min) (IU/L) | 85 (60-127) | 85 (62-122) | .88 |
| ALP (max) (IU/L) | 92 (66-143) | 98 (72-145) | <.001 |
| AST (min) (IU/L) | 39 (24-85) | 29 (19-56) | <.001 |
| AST (max) (IU/L) | 47 (26-116) | 38 (22-81) | <.001 |
| Total bilirubin (min) (mg/dL) | 0.6 (0.4-1.4) | 0.6 (0.4-1.0) | <.001 |
| Total bilirubin (max) (mg/dL) | 0.8 (0.4-1.7) | 0.8 (0.5-1.3) | .80 |

^a^ Data is in count (%) or median (Interquartile range, IQR).

**Abbreviations**

APS III: Acute Physiology Score III

aPTT: activated partial thromboplastin time

ALT: alanine transaminase

ALP: alkaline phosphatase

AST: aspartate aminotransferase

BMI: body mass index

BUN: blood urea nitrogen

CKD: chronic kidney disease

CHF: congestive heart failure

CPD: chronic pulmonary disease

DBP: diastolic blood pressure

eICU-CRD: eICU Collaborative Research Database

GCS: Glasgow Coma Scale

HCT: hematocrit

Hb: hemoglobin

INR: international normalized ratio

MIMIC-IV: Medical Information Mart for Intensive Care database-IV

MAP: mean blood pressure

PLT: platelets

PT: prothrombin time

SOFA: Sequential Organ Failure Assessment

SpO_2_: oxygen saturation

SBP: systolic blood pressure

WBC: white blood cells
