# Supplementary material for: Development and Validation of an Interpretable Conformal Predictor to Predict Sepsis Mortality Risk: Retrospective Cohort Study
Source: J Med Internet Res. 2024 Mar 18;26:e50369. doi: 10.2196/50369 (PMC10985608; doi:10.2196/50369)
Supplement: Multimedia Appendix 2 [file jmir_v26i1e50369_app2.docx]

**Multimedia Appendix 2**

Table 1. Statistical analysis of the top 15 features between the single correct prediction patients and the multiple prediction patients in the eICU-CRD validation populations

| **Variables** | **Survivors** | | |  | **Nonsurvivors** | | |  |  |
| --- | --- | --- | --- | --- | --- | --- | --- | --- | --- |
|  | **Single correct (n=4285, 49.8%)** | **Multiple (n=3323, 38.6%)** | ***P*** |  | **Single correct (n=852, 48.5%)** | **Multiple (n=681, 38.8%)** | ***P*** |  | ***P^a^*** |
| APS III (score), median (IQR) | 41 (32-52) | 58 (45-71) | <.001 |  | 87 (72-107) | 60 (48-74) | <.001 |  | .006 |
| Age (years), median (IQR) | 61 (51-71) | 69 (59-79) | <.001 |  | 71 (61-80) | 71 (60-81) | .98 |  | .007 |
| Urine output (mL), median (IQR) | 1675 (1000-2655) | 1077 (580-1800) | <.001 |  | 400 (140-813) | 903 (400-1493) | <.001 |  | <.001 |
| Vasopressor, n (%) | 801 (18.7) | 1019 (30.7) | <.001 |  | 574 (67.4) | 208 (30.5) | <.001 |  | .95 |
| Pulmonary infection, n (%) | 1675 (39.1) | 2033 (61.2) | <.001 |  | 456 (53.5) | 379 (55.7) | .41 |  | .007 |
| Lactate (min) (mmol/L), median (IQR) | 1.3 (0.9-1.8) | 1.5 (1.1-2.2) | <.001 |  | 2.7 (1.7-4.5) | 1.6 (1.1-2.4) | <.001 |  | .009 |
| BMI (kg/m^2^) , median (IQR) | 28.4 (24.1-34.8) | 26.4 (22.3-31.6) | <.001 |  | 25.7 (21.6-30.6) | 25.7 (22.1-30.6) | .79 |  | .03 |
| WBC count (min) (K/μL), median (IQR) | 10.0 (7.0-13.8) | 11.2 (7.3-15.8) | <.001 |  | 11.7 (6.0-17.4) | 11.9 (7.6-17.0) | .15 |  | .03 |
| SpO_2_ (std) (%), median (IQR) | 1.91 (1.39-2.59) | 2.16 (1.40-3.11) | <.001 |  | 3.09 (1.99-4.64) | 2.30 (1.50-3.17) | <.001 |  | .02 |
| PLT count (max) (K/μL), median (IQR) | 220 (162-296) | 219 (152-311) | .27 |  | 169 (95-271) | 211 (136-310) | <.001 |  | .12 |
| Respiratory rate (mean) (bpm),  median (IQR) | 20 (18-23) | 22 (18-25) | <.001 |  | 24 (20-27) | 23 (19-26) | <.001 |  | <.001 |
| Serum anion gap (min) (mmol/L), median (IQR) | 9 (7-12) | 10 (7-12) | <.001 |  | 12 (9-16) | 10 (8-13) | <.001 |  | .03 |
| BUN (min) (mg/dL), median (IQR) | 17 (10-27) | 25 (15-39) | <.001 |  | 36 (24-53) | 29 (19-45) | <.001 |  | <.001 |
| aPTT (max) (seconds), median (IQR) | 32.4 (28.1-38.0) | 34.6 (29.0-42.8) | <.001 |  | 41.8 (34.0-58.0) | 35.0 (30.9-47.0) | <.001 |  | .01 |
| Serum sodium (max) (mmol/L),  median (IQR) | 140 (137-142) | 140 (137-143) | <.001 |  | 141 (137-145) | 140 (137-144) | .22 |  | .23 |

^a^ Statistical analysis between survival and nonsurvival multiple predictions.

## Abbreviations

APS III: Acute Physiology Score III

aPTT: activated partial thromboplastin time

ALT: alanine transaminase

BMI: body mass index

BUN: blood urea nitrogen

eICU-CRD: eICU Collaborative Research Database

PLT: platelets

WBC: white blood cells
